# Exploring prevention and mitigation strategies to reduce the health impacts of occupational exposure to wildfires for wildland firefighters and related personnel: protocol of a scoping study

**DOI:** 10.1186/s13643-020-01381-y

**Published:** 2020-05-29

**Authors:** Erica Koopmans, Trina Fyfe, Mike Eadie, Chelsea A. Pelletier

**Affiliations:** 1 Health Research Institute, University of Northern British Columbia, Prince George, Canada; 2 Northern Medical Program, University of Northern British Columbia, Prince George, Canada; 3 School of Health Sciences, University of Northern British Columbia, 3333 University Way, Prince George, BC V2N 4Z9 Canada

**Keywords:** Wildfires, Occupational health, Occupational exposure, Mitigation, Prevention, Priority setting, Scoping review, Firefighter, Smoke, Delphi technique

## Abstract

**Background:**

With an increase in wildfire activity across the globe and growing numbers of personnel involved each year, it is necessary to explore the health impacts of occupational exposure to wildfires and the practices and policies that can be implemented to mitigate these effects. The aim of this work is to (1) identify the impact occupational exposure to wildfires has on health outcomes including physical, mental, and social wellbeing; (2) examine the characteristics and effectiveness of mitigation strategies or policies to reduce negative health impacts as reported by current literature and reports; and (3) develop a program of research to address and understand the health impacts of occupational exposure to wildfires based on gaps in the literature and stakeholder priorities.

**Methods:**

This scoping study will be conducted in two phases: (1) scoping literature review and (2) modified Delphi process. The literature review will follow a methodologically rigorous scoping review approach that includes (a) identifying the research question (and protocol development), (b) identifying literature (an iterative process), (c) selecting relevant studies, (d) extracting data into tables, and (e) synthesizing, summarizing, and reporting results. Alongside this, a modified Delphi process will be conducted to define priorities for wildland fire occupational health research. A partnership with the British Columbia (BC) Wildfire Service will enable exploring the appropriateness of identified mitigation strategies and health risks for the BC context.

**Discussion:**

This two-phase approach will provide an in-depth review of the literature of the health impacts of occupational exposure to wildfires and identify mitigation strategies or policies implemented to protect workers and reduce negative health impacts. It is anticipated that these findings may provide recommendations for “quick wins” or initial action that can be implemented within the BC context to reduce negative health outcomes, and inform gaps in context-specific research that needs to be addressed through a strategic, collaborative research program over the next 5 years.

**Systematic review registration:**

Open Science Framework osf.io/ugz4

## Background

As wildfire activity increases internationally, a growing number of personnel are exposed to the health impacts of fighting wildfires. Fire activity continues to escalate due to climate change, land use change, and fire exclusion among other factors. In preparation for more fire activity in the coming years, we must consider what the health impacts of occupational exposure to wildfires are for wildland firefighters and related personnel and explore what practices and policies can be implemented to mitigate risk.

### Background on wildfires and the global context

Across the international wildfire fighting community, there is variation in the nomenclature used for both wildfires and personnel. Broadly, wildfires are defined as an unplanned fire (including human-caused fires) which occurs on forest or range land, grass, brush, scrub, and peat lands, or a prescribed fire that spreads beyond the area authorized for burning [1]. For the purpose of this review, wildfire personnel refers to all individuals who fight these fires and includes type 1 wildland firefighters (also referred to as wildfire fighters, forest fire fighters, or fire rangers), personnel involved with aviation operations, incident management/command teams, and type 2/3 contract crews [1]. Table 1 provides an overview of the definitions of key terms adopted for this review. Regardless of the terminology, climate change, land use change, and cost are commonalities affecting wildfire activity across the globe [2, 3]. Higher temperatures, less precipitation, and earlier snowmelts have led to drier conditions increasing the length of wildfire seasons and the likelihood of more intense long-burning fires [4]. This is evident in the earlier and longer fire seasons faced in countries such as Canada, the USA, Mexico, and Australia [5] and unusual fire activity in areas such as the Amazon rainforest [6] and Swedish arctic forests [7]. Changes in how we use land for industrial, residential, and recreational purposes have also impacted fire activity and suppression efforts [3]. Internationally, the costs associated with wildfire extend beyond the immense expense of fire suppression efforts and include impacts on human life and health, property, and the environment. While the causes and cost of wildfires are similar globally, how these fires are fought varies with differences in local geography and vegetation, crew configurations, and suppression tactics. A brushfire in southeast Australia versus a forest fire in northern British Columbia brings its own unique challenges and indicates a need to look at the specific context to understand local challenges.

**Table 1:** Definitions of key terms adopted for this review

Key term	Definition
Fireline	“The portion of the fire upon which resources are deployed and are actively engaged in the incident. In a general sense, the working area around a fire” [1]
Fire season	“The period(s) of the year where fires are likely to start, spread, and damage values-at-risk sufficient to warrant organized fire suppression; a period of the year set out and commonly referred to in fire prevention legislation” [1]
Type 1 fire crew	“The primary response force consisting of 3 to 21 persons and meet all requirements of the Interagency Exchange Standards” [1]
Type 2 fire crew	“Crews intended for utilization on low to moderate complexity sustained action operations and meet all requirements of the Interagency Exchange Standards” [1]
Type 3 fire crew	“Generally made up of temporary firefighter forces used for mop-up situations that have received some type of basic agency firefighting training” [1]
Wildfire	“An unplanned fire – including unauthorized human-caused fires – occurring on forest or range lands, burning forest vegetation, grass, brush, scrub, peat lands, or a prescribed fire set under regulation which spreads beyond the area authorized for burning” [1]
Wildland	“An area in which development is essentially non-existent, except for roads, railroads, power lines, and similar transportation facilities. Structures, if any, are widely scattered” [1]

### British Columbia (BC) wildfire context

Within Canada, each province and territory is responsible for managing wildfires within their own boundaries. In BC, governance and personnel are managed by the British Columbia Wildfire Service (BCWS). BC is home to approximately 94 million ha of diverse wildland, and across this landscape, the BCWS is confronted with an average of 2000 wildfires each year [8]. The majority of these wildfires are quickly contained by personnel on the ground while larger or difficult to access fires may use fixed-wing or rotary aircraft to assist crews. Recent years have seen an increase in fire activity with an unprecedented amount of land burned, people displaced, and personnel involved. In BC, the peak fire season is typically May to August. The summer of 2017 saw over 1.2 million ha burned with over 4700 personnel engaged in fighting wildfires across BC during peak activity [9]. This included over 2000 contract personnel from the forest industry and over 1200 personnel from outside the province. BC received support from across Canada as well as the USA, Mexico, New Zealand, and Australia [9]. For the first time in BC since 2003, ground personnel from the Canadian Armed Forces were also brought in to fight fires. Historically, this was one of the worst wildfire seasons in BC’s history yet this was followed in 2018 by 2117 fires which consumed 1,354,284 ha of land, surpassing the record of ha burned from 2017 [9]. In 2018, the BCWS engaged 4756 personnel including 1719 contract personnel, 961 out-of-province personnel, and hundreds of staff from the Canadian Armed Forces [9]. In fighting and managing these fires, the BCWS has become an international leader in wildfire management. With an increase in fire activity and growing numbers of personnel involved each year, it is necessary to identify what is known on the health impacts of occupational exposure to wildfires for wildland firefighters and related personnel and identify what practices and policies can be implemented by wildfire agencies to mitigate these effects.

### Existing knowledge on health impacts

A preliminary search was conducted to determine if existing reviews on this topic exist. This search identified four reviews: a critical review of the health impacts of wildfire smoke exposure [10], a review of health effects for both firefighters and the public [11], a systematic review of health impacts from non-occupational exposure [12], and a systematic review examining the health impacts of occupational exposure published in early 2019 [13]. This most recent review reports that research in this field is challenging due to the unpredictability of fire seasons which impacts research plans, and understanding the health impacts faced by wildland firefighters is limited due to the seasonality of work as tracking firefighters for more than one season proves difficult [13]. Much of the research is inconclusive in regard to long-term health impacts [13]. Most research completed to date looks at the cardiovascular or respiratory impacts from wildfire exposure [12, 13], and there is a continued need to look at the health impacts holistically—physical, mental, and emotional. Further, these reviews lack a focus on other personnel beyond firefighters who may also be engaged in wildfire fighting activities such as contract crews and incident management teams. The most recent review searched literature up to January 2017, yet since this time, fire activity has continued to increase with record setting areas burned and increases in “megafires” across the globe, including in countries not typically affected [7]. While these reviews are a starting place in understanding the health impacts of wildfire exposure, they do not identify mitigation strategies or policies implemented to reduce negative health impacts and protect workers. Because wildland fires are typically managed by government agencies, it is reasonable to assume the existence of data reports, policies, and tools that may not be captured in the purely academic search strategy included in most systematic reviews.

### Purpose and objectives

The aim of this work is to (1) identify the impact of occupational exposure to wildfires on health outcomes including physical, mental, and emotional wellbeing and (2) examine the characteristics and effectiveness of prevention/mitigation/management strategies or policies implemented to reduce negative health impacts as reported by current literature and reports. The secondary objective is to use these findings to identify key health research priorities related to occupational exposure to wildfires in the BC context based on gaps in the literature and stakeholder-identified priorities. Using a scoping approach, we will systematically search published and gray literature and conduct a modified Delphi study to answer:

1. What are the occupational health impacts associated with wildland firefighting?
2. What prevention, mitigation, and management strategies or policies have been used to reduce the health impacts of wildland firefighting internationally? What are the characteristics and effectiveness of these approaches?
3. Of the identified strategies, which approaches are contextually relevant to BC and what health issues and mitigation strategies should be given priority for a program of research?

## Methods

This scoping study will be conducted in two phases: (1) scoping literature review and (2) modified Delphi process.

### Project team

This work is being conducted through a collaborative synthesis process with the expertise of the Knowledge Synthesis Centre at the University of Northern British Columbia Health Research Institute. The project team consists of a health sciences researcher and academic lead (CP), knowledge synthesis research associate (EK), health sciences librarian (TF), content expert research associate (ME), and partners within the BCWS. The BCWS will be involved throughout as subject matter experts to ensure the work is relevant and to identify mitigation approaches that hold contextual relevance.

### Phase 1: Literature review

The scoping literature review will provide a broad overview of the research that has been conducted in the area, type of evidence available, and an overview of the main findings. This synthesis methodology examines the extent, range, and nature of research activity in an area to map the field of study and inform new research. Our scoping review methods are informed by the methodologically rigorous scoping review approach described by Arksey and O’Malley [14] and further developed by Levac et al. [15]. This approach includes (a) identifying the research question (and protocol development), (b) identifying literature (an iterative process), (c) selecting relevant studies, (d) extracting data into tables, and (e) synthesizing, summarizing, and reporting results.

#### Identifying the research question and developing the protocol

The preliminary research questions and objectives are presented above. This protocol is reported using the “Preferred Reporting Items for Systematic Review and Meta-Analysis Protocols (PRISMA-P; see additional file 1)” [16, 17]. As a scoping review, our protocol is not eligible for registration in PROSPERO (International Prospective Register of Systematic Reviews) but has been registered in Open Science Framework (OSF; osf.io/ugz4).

##### Inclusion/exclusion criteria

Scoping reviews are typically broad but require defined limits for inclusion and exclusion reflected in our inclusion criteria for the elements of population, concept, context, study design, outcomes, language, and timing.

### Population

This review will focus on publications where the population of interest are individuals who experience occupational exposure to wildfires. This includes wildland firefighters, incident management teams, and aerial firefighters (pilots and air crew personnel); contract personnel or crews from the forest industry; equipment operators; fallers; military; and related personnel at fire bases. Studies that include structural firefighters (city/municipal firefighters) will be included if these structural firefighters are engaged in fighting wildfires, but these studies will be separated out in findings to note prior exposure and differences in equipment utilized and suppression strategies.

### Concept

Each publication must include the concept of wildfires. Wildfires will include unplanned fires occurring on forest or range lands, burning forest vegetation, grass, brush, scrub, and peat lands, and prescribed fires which spread beyond the area authorized for burning. Literature on prescribed burns in controlled situations will be included but separated out recognizing that the stresses and health impacts differ in a controlled situation. It is important to include this literature as health impacts and mitigation strategies may have been developed or initially evaluated in a prescribed burn situation. Inclusion of prescribed burn situations aligns with the government definition of wildfires in BC [1] and the concept of wildfires utilized in a 2019 review by Groot et al. [13].

### Context

In efforts to understand where research on this subject is being conducted presently and varying approaches in risk mitigation, publications will be included regardless of the geographic setting.

### Study design

A wide range of study designs has been selected for inclusion to identify the current scope of research and inform key health research priorities based on gaps in the literature. This review will include studies with quantitative designs (e.g., experimental, quasi-experimental, and observational studies); qualitative studies that use phenomenology, ethnography, grounded theory, and narrative inquiry; and mixed methods studies. Including both quantitative and qualitative data will support a holistic understanding of health issues (e.g., reporting both prevalence and subjective meaning of experiences) that may not be captured by one study methodology alone. Case studies and case reports will be excluded as these are typically produced as single injury reports or learning analyses for workplace injury reporting and it is not feasible to include each individual case study completed for wildfire workplace injuries or fatalities. We will include the following types of publications: academic journal papers, gray literature and reports from the health sector, government, and related sectors. Conference abstracts, editorials, literature reviews/knowledge syntheses, and opinion pieces will be excluded.

### Outcomes

The primary outcomes of interest are (1) occupational health effects of exposure to wildfires and (2) risk management or mitigation strategies, tools, policies, or guidelines to prevent or limit these effects. Occupational health effects include morbidity and mortality outcomes, reported adverse health effects, or problems including physical health, mental health, sleep, fatigue, and stress experienced as a result of work related to fighting wildfires. We will include all eligible studies that report these outcomes. Within these studies, we will explore what, if any risk management and mitigation strategies, policies, or guidelines are discussed. Risk management or mitigation strategies will include tactics, tools, policies, or guidelines implemented by management to prevent or limit occupational health effects of wildfires. Examples of eligible strategies include medical screening, fitness programs, safety training, situational awareness training, standard operating guidelines, staffing, communication tactics, and personal protective equipment [18]. Despite being excluded in previous reviews [13], we have decided to include modeling studies if used as a mitigation strategy or way to track the effectiveness of a strategy against predictions.

### Language

Searches will be limited to publications written in English.

### Timing

Studies will be selected for inclusion from database conception to the date searches are conducted. This will allow us to capture the trend of literature over time and how literature has expanded in this field.

#### Identifying the literature

For this scoping review, search strategies will be created for individual databases with the assistance of a health research librarian and use combinations of subject headings and keywords (see Table 2). A draft search strategy for MEDLINE Ovid is provided in additional file 2. Subject headings and keywords have been identified by reviewing the terms and search strategies used by previously published reviews on the topic and key articles identified by the research team. The following academic databases will be searched (from inception onwards): Ovid MEDLINE, Web of Science, PTSDpubs Proquest, Biological & Agricultural Index Plus EBSCO, CINAHL EBSCO, PsychINFO EBSCO, and GreenFILE EBSCO. Targeted gray literature searches will be undertaken through internet searches of government foresty and wildfire agency websites, industry websites, and work safety and union websites (see additional file 3). References of all papers meeting inclusion criteria will be reviewed for additional papers that have not been identified. We will hand search relevant journals: *Fire*, *Safety*, *International Journal of Wildland Fire*, *Occupational Medicine*, *Journal of Occupational Health*, *International Journal of Occupational Medicine and Environmental Health*, *Environmental Research*, and *Environmental Health Perspectives*. The titles and abstracts of identified articles will be uploaded into Distiller SR systematic review software (Evidence Partners, Ottawa, Canada) for screening.

**Table 2:** Keywords to be utilized in search strategies

Concept	Text words
Wildfire	fire, wildfire, wild fire, forest fire, brush fire, brushfire, wildland fire, bushfire, grassfire, prescribed burn, prescribed fire, smoke pollution, smoke, wood, simulated
Personnel and crew configurations	Firefighter, project firefighter, wildland firefighter, wildfire firefighter, wildfire fighter, forest firefighter, fire ranger, unit crew, handcrew, hotshots, project crew, initial attack, heliattack, helitack, rapattack, rappel crew, parattack, smokejumpers, engine crew, air crew, aerial firefighters, fire base, contract crews, military, forest industry, occupation, suppression
Health outcomes	Health, exposure, casualty, death, disease, illness, morbidity, mortality, effect, adverse effect, impact, health problem, health hazard, mental health, sleep, fatigue, stress, shift work
Intervention or mitigation	Mitigation, intervention, prevention, policy, guidelines

#### Selecting relevant publications

In DistillerSR (Evidence Partners, Ottawa, Canada), all peer-reviewed publications as well as documents from the gray literature that examine health impacts of occupational exposure to wildfires will be screened for inclusion or exclusion according to the predetermined eligibility criteria. Screening will occur in two stages: (1) title and abstract and (2) full text. Two reviewers will independently screen the titles and abstracts of search results using the eligibility criteria. An initial calibration screening of 10% of the articles will be conducted to ensure reliability in correctly selecting articles for inclusion and to assess inter-rater agreement. Potentially relevant publications will continue to the next stage where the full text is retrieved and independently reviewed by two reviewers using the same eligibility criteria. Disagreements regarding inclusion will be resolved through a third reviewer. Reasons for exclusion and any deviations from the planned protocol will be recorded.

#### Extracting the data

A data extraction template created in DistillerSR (see additional file 4) will be used to extract study details including author, year, journal, country of origin, study design, and participants. We will also extract information on the health outcomes that have been assessed, the main findings, conclusions, and recommendations for mitigation and/or policy as outlined by current literature. The data extraction template will be piloted for use on five articles, reviewed by the study team, and then revised as needed. Data extraction will be completed independently by a first reviewer, and a second reviewer will complete “quality control” or check their work for accuracy. If the second reviewer does not agree with the initial review, it will be flagged by DistillerSR for conflict resolution by a third team member.

#### Synthesizing, summarizing, and reporting the results

We will synthesize peer-reviewed publications and gray literature using a narrative descriptive summary and descriptive tables to summarize the number of studies by broad categories (e.g., health outcome or mitigation strategy) and subgroups (e.g., mental health outcomes). Depending on the breadth of studies identified, we may also categorize papers based on study design (e.g., prospective, retrospective, cross-sectional) and location of study. Additionally, we will utilize tabular and graphical representations to summarize publications, their characteristics, the health outcomes assessed, and main findings. Consistent with a scoping review approach [14, 15], we will not assess study quality or risk of bias. Because the objective of this project is to broadly map and identify all available evidence from both academic and gray literature, we will not exclude any identified studies based on quality.

### Phase 2: Delphi study

The Delphi method is a flexible iterative process used to achieve consensus and is commonly used to identify research priorities [19–23]. This allows stakeholders to express their knowledge, provide feedback, and consider the viewpoints of others as they develop opinions. There are many models of this approach, the most common involves conducting anonymous surveys to collect data, analyzing data, and presenting data back to all participants for consideration and reassessment. Each step is considered a “round” and can be repeated a number of times depending on if researchers are interested in achieving consensus. Following a modified Delphi approach, we will use an online survey and ask knowledge user stakeholders to list up to 10 research priorities or topic areas for wildland firefighter health research over the next 5 years (round 1). We will complete a thematic analysis of responses to the first round survey to develop research topics. As a research team, and in consultation with stakeholder partners at the BCWS, we will discuss codes and themes as they are identified to reach consensus on a list of topics and subtopics. In the second round, we will ask participants to rank the importance of each identified priority (topic) using a 5-point Likert scale [24]. The order that each topic will be presented to each participant will be randomized. Although we will not impose a strict limit on the number of research topics to propose for the second round survey, we will limit the number presented to 20 to ensure the second survey is not too long or onerous for participants. The final page of the survey will include a complete list of the topics, and participants will be asked to rank-order the research topics based on urgency, relevance, and importance. Consensus will be determined if more than 70% of participants indicate they strongly agree or agree that a topic is a priority or if they disagree or strongly disagree that a specific topic is a priority [25]. Research topics will be organized by role/occupation of participants (e.g., wildland firefighter, incident management team member, researcher) and based on identified priority rank (e.g., number of people ranking a specific topic as strongly agree/agree). Based on previous research, we anticipate two rounds will be sufficient to establish consensus on research priorities [26–28]. The online process will enable a broad number of participants from various stakeholder groups to participate without imposing geographical constraints.

Meetings with researchers and similar service organizations provincially, nationally, and/or globally may also be conducted to understand current work and mitigation strategies that may not be reported in published literature. These consultations and the Delphi questionnaire will help to identify strategies, determine which are contextually relevant or need to be adapted for use in BC, and what mitigation strategies and health issues should be given priority for a program of research.

Research ethics board approval has been obtained from the University of Northern British Columbia for phase 2 (modified Delphi study) of this project. Informed consent will be obtained from all participants.

## Discussion

This scoping review will identify and map evidence related to the health risks of occupational exposure to wildfire and review the effectiveness of mitigation strategies and/or policies that have been implemented. Combined with a modified Delphi process, we will use identified gaps in the literature to develop a stakeholder-driven program of research that can be implemented in the BC context and help to guide international research efforts. With the increasing prevalence, severity, and impact of wildland fire, this area of research is emerging as an urgent priority. This project will provide an important collation of academic and gray literature on this topic and develop a list of research priorities based on gaps in the literature and stakeholder priorities.

### Strengths and limitations

The key strength of this protocol is the use of rigorous, transparent, and systematic methods in the selection, analysis, and synthesis of available literature. We have outlined a search strategy for both academic and gray literature designed by a health sciences librarian that will provide a comprehensive review of the field as applicable to policy and practice. This is complemented by the collaborative approach with key informants working in the sector with experience of the perceived and evident health impacts of exposure to provide contextualization to the findings. The major limitation of this review is the inclusion of publications written only in English. We recognize that by limiting to the English language only we may miss other pertinent publications. For example, there may be Spanish and/or Portuguese publications missed from countries such as Mexico and Portugal which encounter many wildfires. A second limitation is the exclusion of case studies or case reports. It is possible these reports may have useful insights on lessons learned from individual events; however, it is not feasible to include each individual case study completed for wildfire workplace injuries or fatalities. Finally, this method of review is focused on breadth or scope of research rather than depth (quality) and provides a narrative or descriptive account of the published research. Scoping review methodology does not include critical appraisal of the quality of evidence or assessment of risk of bias in the primary research reports which may limit confidence and validity of our study findings.

### Implications and recommendations

This project will provide an in-depth review of literature with synthesis of the health impacts of occupational exposure to wildfires and mitigation strategies or policies implemented to protect workers. It is anticipated that these findings may provide recommendations for “quick wins” or initial action that can be implemented locally to reduce negative health outcomes, and inform gaps in knowledge to be addressed through a strategic, collaborative research program over the next 5 years. This project will have relevance for researchers, government agencies that employ and manage wildland firefighters and related personnel, health and safety regulatory agencies, and people who are employed in wildland fire suppression. The results of this review will be published in a peer-reviewed journal and disseminated at relevant conferences. The proposed scoping review will be reported in accordance with the reporting guidance provided in the PRISMA extension for Scoping Reviews (PRISMA-ScR) [29]. Any important protocol amendments will be documented on the OSF webpage (https://osf.io/ugz4s) and reported in the final publication on findings. We will also prepare a plain language summary and present the review findings to local stakeholders in BC, including the BCWS and work safety representatives. Partner wildfire organizations will support dissemination through wider networks across Canada and internationally.

## Supplementary information

**Additional file 1.** PRISMA-P Checklist.

**Additional file 2.** Literature Search Strategy.

**Additional file 3.** Gray Literature Search Strategy.

**Additional file 4.** Data Extraction Template.

## Data Availability

Not applicable.

## Abbreviations

BC: British Columbia
BCWS: British Columbia Wildfire Service
PRISMA-P: Preferred Reporting Items for Systematic Reviews and Meta-Analysis Protocols
OSF: Open Science Framework
