# Supplementary material for: Exploring prevention and mitigation strategies to reduce the health impacts of occupational exposure to wildfires for wildland firefighters and related personnel: protocol of a scoping study
Source: Syst Rev. 2020 May 29;9:119. doi: 10.1186/s13643-020-01381-y (PMC7257175; doi:10.1186/s13643-020-01381-y)
Supplement: Supplementary file 3 — Additional file 3. Gray Literature Search Strategy. [file 13643_2020_1381_MOESM3_ESM.docx]

1. **Identification of relevant authority stakeholders**
   1. Identify wildfire agencies internationally with assistance of content experts from BCWS (limited to English speaking countries)

| **Country** | **Agencies** |
| --- | --- |
| Canada | - [Canadian Interagency Forest Fire Centre](https://www.ciffc.ca/) - [BC Wildfire Service](https://www2.gov.bc.ca/gov/content/safety/wildfire-status) - [Alberta Wildfire](https://wildfire.alberta.ca/) - [Government of Saskatchewan Wildfire Operations](https://www.saskatchewan.ca/fire#utm_campaign=q2_2015&utm_medium=short&utm_source=%2Ffire) - [Manitoba Sustainable Development Wildfire Program](https://www.gov.mb.ca/wildfire/index.html) - Ontario [Ministry of Natural Resources and Forestry](https://www.ontario.ca/page/forest-fires) - Quebec [Société de protection des forêts contre le feu (SOPFEU)](https://sopfeu.qc.ca/en/) - Nova Scotia [Department of Natural Resources Wildfire Management](https://novascotia.ca/natr/forestprotection/wildfire/) - Yukon [Wildland Fire Management](https://yukon.ca/en/get-wildfire-updates) - Northwest Territories [Wildland Fire Operations](https://www.enr.gov.nt.ca/en/services/wildfire-operations) |
| United States | - [U.S. Forest Service](https://www.fs.fed.us/science-technology/fire/people) |
| Australia | - [Australasian Fire Authorities Council](https://www.afac.com.au/) - [Bushfire and Natural Hazards CRC](https://www.bnhcrc.com.au/research) - [Government of New South Wales – New South Wales Rural Fire Service](https://www.rfs.nsw.gov.au/about-us/employment) - [Queensland Fire and Emergency Services - Rural Fire Service](https://www.qfes.qld.gov.au/employment/jobs/rural-fire-service-volunteer.html) - [South Australian Fire and Emergency Services Commission – Country Fire Service](https://www.environment.sa.gov.au/topics/fire-management/project-firefighters) - [Tasmania Fire Service](http://www.fire.tas.gov.au/Show?pageId=colServices) - [Department of Environment, Land, Water, and Planning](https://www.ffm.vic.gov.au/) - [Department of Fire and Emergency Services of Western Australia](https://www.dfes.wa.gov.au/waemergencyandriskmanagement/obrm/Pages/default.aspx) |
| New Zealand | - [Fire and Emergency New Zealand](http://www.fire.org.nz/about-us/what-we-do/) |

1. **Search of agency websites for reports, documents, protocols, and guidelines using key terms on health, occupational health, exposure, mitigation, intervention, prevention, policy, and guidelines. The date, agency searched, hyperlink, and relevant documents identified will be documented.**
2. **Contact experts and authors in this field of research to find out about studies or reports we may have missed and additional sources of grey literature**
