# Supplementary material for: Exploring prevention and mitigation strategies to reduce the health impacts of occupational exposure to wildfires for wildland firefighters and related personnel: protocol of a scoping study
Source: Syst Rev. 2020 May 29;9:119. doi: 10.1186/s13643-020-01381-y (PMC7257175; doi:10.1186/s13643-020-01381-y)
Supplement: Supplementary file 4 — Additional file 4. Data Extraction Template. [file 13643_2020_1381_MOESM4_ESM.docx]

| **Items to extract** | **Extracted Info** |
| --- | --- |
| Author(s) |  |
| Title |  |
| Year of publication |  |
| Journal |  |
| Author institutional affiliations |  |
| Country of study origin |  |
| Setting (geographic setting) |  |
| Study Design |  |
| Study Population |  |
| Sample Size |  |
| Aims, objectives, or purpose |  |
| Health outcomes assessed/measured |  |
| Main Findings |  |
| Recommendations, strategies, policies, or tools for prevention or mitigation to reduce health impacts of occupational exposure |  |
| Conclusions |  |
| Author disclosures/conflicts of interest |  |
